# Estimation of Linear Models from Coarsened Observations: A Method of Moments Approach

**DOI:** 10.1017/psy.2024.19

**Published:** 2025-03-10

**Authors:** Bernard M. S. van Praag, J. Peter Hop, William H. Greene

**Affiliations:** 1 Tinbergen Institute, University of Amsterdam, The Netherlands; 2 Independent, The Netherlands; 3 University of South Florida, USA

**Keywords:** coarsened events, generalized method of moments, item response models, multivariate ordered probit, ordered qualitative data, ordinal data analysis, polychoric correlations

## Abstract

In the last few decades, the study of ordinal data in which the variable of interest is not exactly observed but only known to be in a specific ordinal category has become important. To emphasize that the problem is not specific to a specific discipline we will use the neutral term coarsened observation. For single-equation models estimation of the latent linear model by Maximum Likelihood (ML) is routine. But, for higher-dimensional multivariate models it is computationally cumbersome as estimation requires the evaluation of multivariate normal distribution functions on a large scale. Our proposed alternative estimation method, based on the Generalized Method of Moments (GMM), circumvents this multivariate integration problem. It can be implemented by repeated application of standard techniques and provides a simpler and faster approach than the usual ML approach. It is applicable to multiple-equation models with 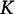 -dimensional error correlation matrices and 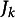 response categories for the kth equation. It also yields a simple method to estimate polyserial and polychoric correlations. Comparison of our method with the outcomes of the Stata ML procedure cmp yields estimates that are not statistically different, while estimation by our method requires only a fraction of the computing time.

## 1 Introduction

The statistical tools of empirical Psychometrics, Econometrics, Political Science, and many other empirical sciences including marketing analysis, agriculture, health, and medical statistics, find their origin in the linear regression model.^1^ The idea is that a random phenomenon *Y* can be *predicted* by variables *X* in the sense that 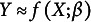 , where 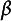 is a parameter vector. Later, the same model is applied to *explain* the phenomenon *Y*, as caused by the variables *X.* In the econometrics literature since the 1960s this resulted in a host of different models, described in textbooks such as Greene (2018) and Cameron and Trivedi (2005). In psychometrics there was a similar development known as the Structural Equation Model (SEM) (Duncan, 1975; Hayduk, 1987; Bollen, 1989; Jöreskog, 1973). (For software, see, for example, Narayanan (2012).) The main idea behind the modeling approach is that the phenomenon 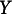 to be studied has a conditional mean function that depends on other variables 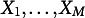 , say, 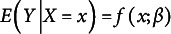 , where 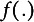 is a continuous and differentiable function and 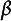 is a set of parameters. In practice the model is usually taken to be linear, that is, 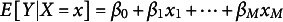 , which produces the linear regression model. In the traditional approach, the variable 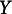 is continuous and directly observable. In economics the model approach (the first influential introduction is by Hood and Koopmans (1953)) is used to describe dependencies between economic variables, e.g., purchase intentions as a function of income, prices, education, family size, and age. In marketing the modeling approach is used to develop and assess the effects of advertising, prices, promotions, etc. (see e.g., Fok (2017)). In medical statistics, a model is used to evaluate the response to diagnostic tests. These are a few examples to illustrate the pervasiveness of the regression model approach in empirical sciences.

The one-dimensional observation 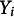 may be replaced by a 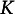 -dimensional vector, 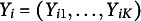 and the function 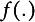 by a 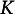 -vector function, 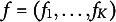 .

In practice, the parameters of interest in such a model are estimated from a set of 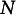 observations 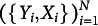 . Randomness enters the observed outcomes through the difference between the individual responses, 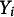 , and the conditional expectation for individual observation 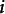 , 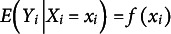 . Hence, we introduce an “error,” 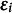 , or a 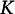 -vector of errors, 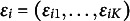 , which is defined for each response as 
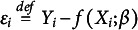
 . The error represents the aggregate of other possible, unobserved variables as well as the randomness of individual behavior. The random term is generated by a mean zero process that operates stochastically independently of 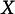 . If the model is linear, we denote the discrepancy as the residual 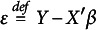 . This approach is applicable when the dependent variable(s) 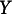 and the explanatory variable(s) 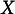 is (are) cardinal, i.e., are expressed in observable numerical values.

However, in many practical cases the dependent variable 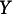 is coarsened; it is only observable in terms of ordinal categories on a preference scale, such as subjective health status, well-being, reported as “bad” or “good,” or “like” or “dislike,” or “poor, fair, good, very good, excellent,” or some ordinal ranking from one to five where a cardinal interpretation becomes dubious. Although the observations take place in a coarsened mode, we must interpret the discrete answers as reflecting a latent variable 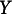 , the range of which is a continuum. In that situation, we say the observations are “coarsened” or condensed into a set of 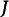 adjacent intervals 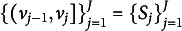 on the real axis. For an individual *i*, the latent observation is 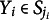 , if the realized observation is 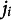 . Hence, we see that the estimation of the model is complicated by two factors. First, there is the statistical problem that there is always a random error term involved. Second, there is the additional observation problem that the continuum of observations is coarsened or condensed (Maris, 1995) and mapped on a discrete event space 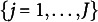 . We will call such data Ordinally Coarsened (OC).

Since about 1934 in bioassay studies (Bliss, 1934; Finney, 1947, 1971), and in Economics, Sociology, and Political Science, much later in the 1960s and 1970s researchers realized that many variables of interest have an ordinal coarsened character. For instance, a question on self-assessed health status may be responded to with ordered labels varying from “very healthy,” to “very unhealthy.” In modern datasets, especially survey data, such verbal evaluations are abundant. The World Happiness Report, Oxford University (2024) is a notable example. Such coarsening is nearly always dictated by the fact that respondents are unable to quantify their answers directly on a numerical continuous scale but only in terms of a few ordered verbally described categories. These values are not expressed in numbers but in ordinal qualitative terms. In Psychometrics popular item response theory offers many examples (see Wiberg et al. (2019)). To stress that the statistical problems are not specific to one discipline we will also speak of coarsened data.

In psychometric Structural Equations models (SEM) things may be further complicated by the fact that the explanatory 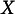 variables are sometimes coarsened as well (cf. Jöreskog (1973)). In this paper, we will assume that the explanatory 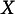 variables are either directly observable on a continuous scale, or coarsened in ordered classes labeled 1, 2,.., or by dichotomous dummy variables, taking the value zero or one.

A specific case of coarsened events is that of psychological testing (item-response theory IRT) and that of multiple-choice tests used in exams. Say, the exam or test consists of *K* items with each *J ordered* response categories. Then the test may be described by *K* item scores 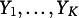 . The response to each separate item may be dichotomous (e.g. correct/false) or polychotomous (correct, not wholly correct,…, false).

The probability of the response on item *k* is 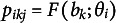 , where 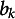 stands for the difficulty of the item *k* and 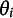 for the ability of respondent *i.* From the joint probability of the *K* responses by individual *i* we try to estimate the latent ability 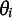 (e.g., IQ) of the individual by maximizing per individual *i* the joint probability 
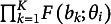
 , with respect to 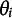 . The ability 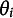 may also depend on (or be explained by) individual observable characteristics 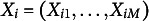 , say 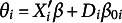 ,where the dummy variable 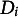 equals one for individual *i* and zero for others and 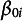 is the ability of individual *i*. The term 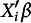 gives then the part of ability or intelligence that can be explained by e.g., education, genetic factors, health, income, etc., while the individual parameter 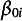 may be identified as the unexplainable truly individual ability component. We notice that the estimation of 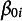 requires that *K > 1* and preferably considerably larger than one.

The method developed below may be used to estimate these 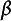 ’s and 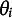 ’s. The response probability 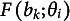 of a separate item score is frequently assumed to be described by a normal or logistic distribution function. Estimation of 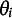 is then rather easy. However, intuition tells us that mostly, the item scores on different items by a respondent will be correlated. This is almost unavoidable if the response behavior depends on one 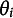 . In that case, the ML- estimation is mostly difficult since estimation will require the evaluation of many multivariate integrals. The correlation means that the multivariate integrals cannot be reduced to products of one-dimensional integrals. The method proposed by us will avoid this problem.

For directly observed cardinal data, ordinary least squares (OLS) is usually the default estimator of choice.^2^ There are many extensions of the OLS estimator that are used in nonstandard cases, such as nonzero covariances across observations. A familiar alternative to OLS is Generalized Least Squares (GLS), in which the disturbances of the 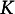 observations per observation unit 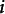 are heteroskedastic or correlated. Then an unknown error-covariance matrix has to be estimated as well. If this is feasible, we call the method Feasible Generalized Least Squares (FGLS). Another well-known example is Seemingly Unrelated Regressions (SUR), where 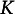 response variables are explained by 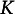 equations where the errors are correlated. We refer to well-known econometric textbooks such as Amemiya (1985), Cameron and Trivedi (2005), Greene (2018) and Verbeek (2017) for elaborate descriptions. We note that in modern work these estimations are usually based on the assumption of a known error distribution, usually multivariate normal, leading to Maximum Likelihood (ML) estimation. In the early received literature, least squares were not explicitly based on an underlying error distribution, but rather on the minimization of a Sum of Squared Residuals (SSR) that led to unbiased estimation of the regression coefficients. Later it was found that SSR minimization and ML-estimation led to the same estimator when the errors are normally distributed. (The normality assumption was also used to motivate certain inference procedures.) The common counterpart for the linear regression model in case of coarsening of 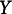 is the Ordered Probit or Ordered Logit model (OP or OL). In the literature, it is frequently called Probit or Logit Regression. (See e.g., McKelvey and Zavoina (1975)).

In this paper we will develop a novel approach to coarsened data, called Feasible Multivariate Ordered Probit (FMOP), where the errors are suspected of being correlated, as is the case in, e.g., item response models, in panel data, regional data or customer satisfaction data. It follows the analogy principle as formulated by Goldberger (1968, 1991) and Manski (1988), based on the method of moments (MoM).

The established way to treat such models with coarsened correlated observations in psychometrics or econometrics, and other empirical applications is by maximum likelihood (ML) estimation (see McFadden (1989) and Hajivassiliou and McFadden (1998)), where the likelihoods per observation include 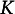 -variate normal integrals (instead of densities). Those integrals are generally estimated by simulation such as by the Geweke-Hajivasilliou-Kean (GHK)-algorithm. Theoretically, this is a straightforward application of ML theory. However, the practical problem is that the evaluation of those integrals by simulation may be quite cumbersome and time-consuming.

Geweke (1989), McFadden (1989), Keane (1994), Hajivassiliou and McFadden (1998), Cappellari and Jenkins (2003) and Mullahy (2016), and others developed a multivariate probit estimator. Progress has also been made based on the simulated moments approach (McFadden, 1989), using the Gibbs sampler method (see Geman and Geman (1984) and Casella and George (1992)). An interesting historical survey on the (logit and) probit method is found in Cramer (2004). See also Hensher et al. (2015). Roodman (2007, 2020) developed a flexible working Stata estimation procedure (cmp) based on the GHK simulator.

Independently, scholars in Psychometrics expanded the vast literature on IRT models yielding different tools of analysis, inspired by the differences between the subject matters between disciplines (see Bollen (1989)). It is surprising that both psychometricians and econometricians are working on essentially the same methodological problems, but mostly without taking much notice of each other’s literature. A rare exception is the econometrician Goldberger (1971) who explicitly recognized the commonalities between Econometrics and Psychometrics.

Our approach, based on sample moments, does not need the evaluation of likelihoods, i.e., multi-dimensional probability integrals or simulated moments. In this paper, we assume multivariate normal error distributions for 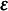 in line with the established practice. Data on 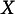 are assumed to be generated by a random process that is statistically (and functionally) independent of that which generates 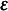 . The important element is that the observed values of 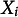 convey no information about the errors 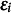 , a property identified by econometricians as “strict exogeneity.” It is the property that 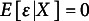 for every 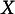 . It follows that 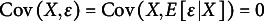 as well. Econometricians call this condition strict exogeneity. It implies the same zero-covariance property. Data on 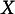 are assumed to be “well-behaved,” meaning that in any random sample, the sample covariance matrix 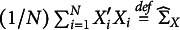 is always finite and positive definite. (Regularity conditions on *X* such as that the influence of any individual 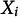 in 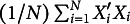 vanishes as 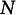 increases are also assumed.) Nothing further is assumed at this point about the distribution of 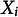 , e.g., normality, discreteness, etc.

The theoretical model, that is, the data-generating mechanism, mimics the classical linear statistical models. The substantive difference between exact and coarsened data is in the mode of observation of the dependent variable 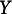 . In the classical framework the dependent variable 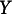 is directly observed, while in the Ordered Coarsened (OC) -observation mode, the dependent variable 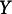 is only observed to be in one of the *J* intervals 
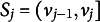
 , where the cut points 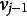 and 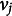 are unknown parameters to be estimated. If 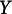 is a *K-*vector, 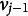 and 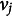 are *K-*vectors and 
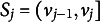
 is a block in 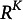 . Since the cut points 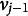 and 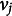 are unknown, it follows that the unit of measurement of 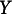 is unidentified. The usual identification is secured by setting the error variances equal to one; 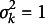 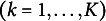 .^3^

The structure of this paper is as follows. In Section 2 we outline the basic probabilistic model in the presence of coarsening of the dependent variables. In Section 3 we develop the estimation method for a 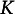 *-*equations model based on the Ordered Coarsened data model. In IRT-models, this is equivalent to 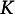 item responses per respondent. We call the method Feasible Multivariate Ordered Probit (FMOP). We call it Seemingly Unrelated Ordered Probit (SUOP) when we have a 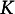 *-*equation model with one observation 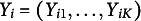 per observation unit *i*. Of course, mixtures of FMOP and SUOP are possible as well. Instead of differentiating high-dimensional log-likelihoods, with the likelihoods being multi-dimensional integrals, with respect to the unknown parameters 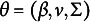 , we derive sample moment conditions 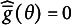 from the coarsened data that are analogues of the likelihood equations, 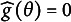 for direct observations. We then estimate 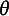 from the equation set 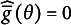 . We find that 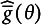 and 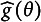 converge to the same probability limit for all values of 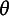 . The estimation of 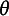 from 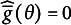 takes place by repeatedly applying the generalized method of moments (GMM), (Hansen, 1982). We note in passing that our approach employs elements of the EM algorithm (Dempster et al., 1977). In Section 4 we demonstrate how to estimate polychoric correlations from SUOP-estimates. Further, we generalize the concept of the coefficient of determination, 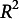 , to 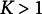 . In Section 5.1 we apply FMOP to an employment equation over a five-year panel dataset from the German SOEP dataset. In Section 5.2 we apply SUOP to a block of eight satisfaction questions extracted from the German SOEP data. In order to get more insight into the stability of the method, in Section 5.3. we do some experiments on a simulated data set. We use a recent update of the Stata procedure cmp as our benchmark to compare our alternative approach with the ML approach.

We find that the estimation results of regression coefficients and their standard errors do not differ substantially between the two methods. Where the established method may need hours, our method takes only minutes. In Section 6 we provide some concluding remarks. In the Appendix, we propose an easy intuitively appealing method to estimate the latent full error-covariance matrix, after the regression coefficients, 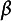 , have been estimated using the coarsened data.

## 2 Regression in the population space

Regression analysis often begins with assumptions about the distribution of the observed data. Each estimation procedure on a sample may be seen as a reflection of a similar procedure in the population. For convenience but without loss of generality we assume 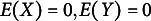 . The model of interest for equation *k* of 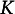 equations is
(2.1)

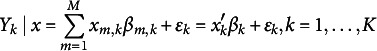



Where the model design might call for differences across equations, they can be accommodated by suitable zero restrictions on the coefficients in 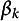 . For convenience, we ignore constant terms by setting 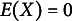 . The expectation of outcome or response 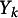 is conditioned or co-determined by explanatory variables/stimuli 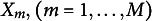 assuming values 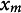 . There are 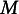 variables 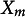 , that are generated by a strictly exogenous process. The error vector 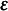 derives from observation-specific variation around the theoretical conditional mean. We have 
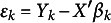
 . Denoting the observations by 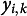 the observed residual is defined as 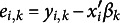 . Deviations of observations 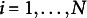 of 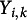 from the conditional mean result from the presence of unobserved elements that enter the data-generating process, for example, variation across individuals in the self-assessment of health or well-being. Random elements are assumed to be generated by a zero mean, finite variance process; if 
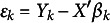
 , it follows that 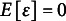 and 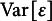 is finite. We assume that the process that generates 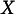 is stochastically independent of that of 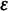 . This implies 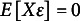 . Substitution yields the familiar regression equations
(2.2)

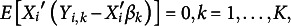

 where 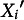 is a 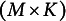 -matrix and 
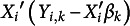
 a *K*-vector.

If this holds for all 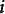 , then we have
(2.3)

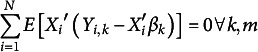

 from which the regression coefficients 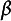 can be solved.

We define the functions 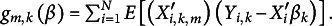 The equation system (2.3) in 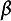 is then shortly written as 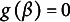 .

Result (2.3) identifies the slopes 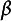 of the conditional mean function. The zero conditional mean result in (2.2) motivates least squares without reference to minimizing the mean squared error prediction of 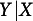 or minimum variance linear unbiased estimation of 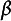 .

If the error covariance matrix 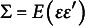 is not the identity matrix, we may want to correct for the unequal variances and correlations, and we weigh the observations by 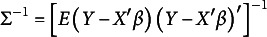 , producing

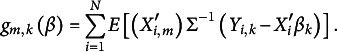



By weighting the 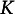 observations per individual *i* by 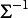 variances are standardized, and the error correlations are accounted for. In that case, according to the Aitken theorem the covariance matrix of the estimator 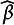 is minimized.

We have motivated least squares through the moment equations (2.2). We see that we can interpret these conditions (2.2) as first-order conditions for minimizing the expectation of the squared residuals 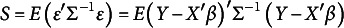 .

We do not need to specify the probability distribution of 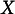 and *Y*. We do assume well- behaved data generating processes, which will include a finite, positive variance of 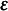 and a finite positive covariance matrix, 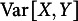 . If the marginal probability distribution of 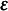 is multivariate normal, the regression estimator is Maximum Likelihood. We call 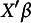 the structural part of the model and 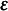 the disturbance, where 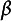 is the 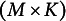 – matrix with columns 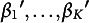 .

If the columns of the matrix 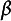 are identical, this is the typical setting for longitudinal and panel data. If the coefficients vary by response setting 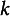 , 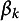 we have the situation of 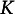 different model equations.

### 2.1 Regression for Coarsened observations

We call an observation 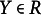 coarsened if it is not observed directly, but only as belonging to one of the 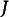 intervals 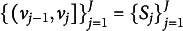 (The leftmost and rightmost terminals are infinity). These intervals constitute the class ℂ of observable events. We will call ℂ the observation grid. This is generalized to 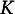 -dimensional observations by replacing the *J* observed intervals 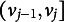 by 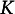 -dimensional blocks.

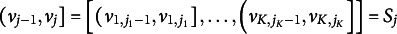

where the one-dimensional random observations 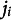 are replaced by the index vectors 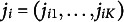 . ℂ stands for a partition in 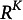 consisting of 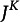 adjacent blocks. (We take 
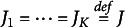
 for convenience, but equality is not necessary.) We denote the ℂ-coarsened event space by 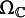 . We may then define the corresponding coarsened probability measure 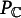 on ℂ by 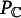 . We will call ℂ the 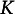 -dimensional observation grid.

Coarsening of 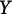 implies that we do not directly observe 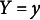 , but the event 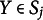 , and more explicitly for a 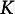 -vector 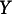 that 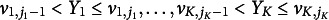 . It follows that for given 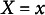 and 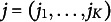 there holds for the error vector

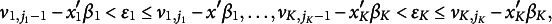

 which we denote shortly as 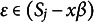 . We denote the marginal probability as 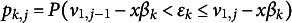 . We define the generalized residual as 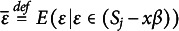 . It is a random 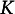 *-*vector defined on the blocks 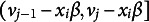 . These blocks constitute individual *i’s* individualized observation grid ℂ 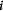 . The grid ℂ 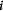 for observation unit 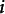 depends on 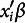 . However, for any given value 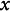 of 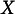 and any grid ℂ 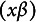 we have 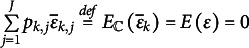 according to the Law of Iterated Expectations(LIE). Then it follows that
(2.4)

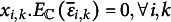

 and
(2.5)

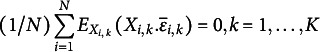



This is the coarsened analogue of (2.2). We define the function 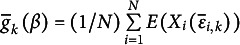 . The equation system (2.4) is then shortly written as 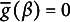 . If the error covariance matrix is not the identity matrix, we may want to correct for the unequal variances and correlations, and we write 
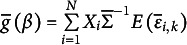
 , where 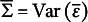 .

Finally, we have for the two functions
(2.6)

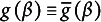



This holds for all values of 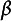 , not only for the zero roots of (2.2) and (2.4). (2.5) holds since for any 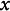 - value 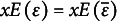 .

## 3 Large sample results for regression

The Law of Large Numbers states that under standard regularity conditions, sample moments converge in probability to their population counterparts as the number 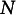 of observations grows large. Slutsky’s theorem says that continuous and differentiable functions of random sample moments converge in probability to those functions of the population counterparts as 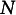 grows large, implying that the population functions 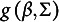 are consistently estimated by filling in the corresponding sample moments.

When we want to get its (large-)sample estimator 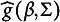 we replace the expectations in (2.2) with the corresponding sample moment conditions and we get
(3.1)

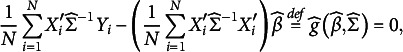

 where 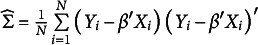 . Solution of (3.1) with respect to the regression coefficients 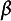 yields
(3.2)

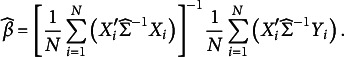



This is the well-known OLS- estimator.

Estimation of the asymptotic covariance matrix 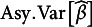 is usually understood to mean under the condition that 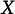 equals the sample data 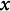 . Then the well-known template is
(3.3)

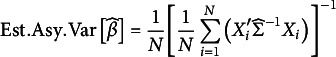



### 3.1 Estimation from ordered coarsened data

Let us now consider the coarsened analogue. The events are elements of the observation grid ℂ. The corresponding coarsened probability measure is *P*_ℂ_. If we would follow the conditional ML strategy, the information to be maximized is *E*_ℂ_ (ln *P*_ℂ_) = 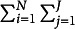 *P*_ℂ_ 
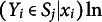
 *P*_ℂ_ 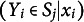 .

The problem is here the evaluation of the *P*_ℂ_ 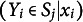 , being multivariate integrals over rectangular blocks in 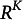 . We can evaluate *P*_ℂ_ 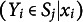 by its sample analogue, but this entails the evaluation of a multitude of 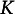 -dimensional integrals, making this procedure very cumbersome, albeit not impossible (see Roodman (2011)).

A much easier way is by making a detour and evaluating the coarsened analogue of the condition (3.1). Let 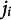 be the 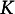 -dimensional response by individual *i.* We notice that (the 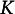 -dimensional) 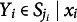 implies 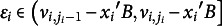 . In this way to each observation unit 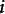 is assigned its own observation grid ℂ*_i_* depending on 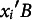 . We define the *K-*vector of the generalized residuals 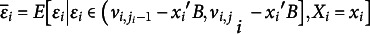 .

The grid ℂ*_i_* over which the expectation is taken at the LHS in (3.1) is now a grid in the space 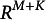 where the first 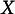 -coordinates are directly observed while the *K* 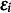 -coordinates are coarsened by ℂ*_i_*. Summing over the observations we get.
(3.4)

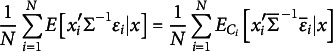



Then (3.4) may be summarized as the identity
(3.5)

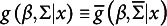



This implies that the equations 
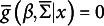
 and 
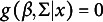
 have the same roots 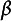 . The vector function 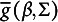 may be interpreted as the vector of derivatives of a criterion function like a log-likelihood or the sum of squared residuals with respect to 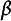 . The simplest criterion function with 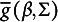 as gradient vector^4^ is

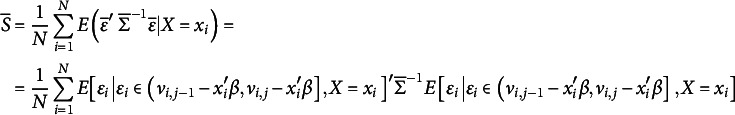



This is the sum of Squared Generalized Residuals. The identity (3.5) implies that 
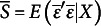
 and 
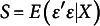
 have the same derivatives with respect to 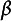 ; consequently, they are identical except for a constant. When we decompose the residual variance into the sum of between- and within-variance 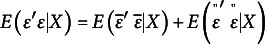 , it is obvious that this constant difference is just the within-variance 
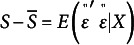
 , which appears not to depend on 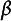 . Things are complicated since each individual 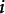 has its own observation grid ℂ*_i_*.

The solution for 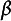 is found as the root vector of the 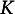 -equation system 
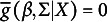
 .

We have now to construct the sample analogue of 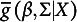 . The 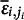 have not drawn much attention in the empirical literature. One notable exception is Heckman (1976) who appears to be the first author in econometric literature to recognize the importance of this expected residual, later in the econometric literature sometimes called the Heckman-term (see also Van de Ven and Van Praag (1981)). They have been called by Gouriéroux et al. (1987) the generalized residuals. They used them in the analysis of residuals. If the exact errors 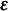 are standard normally distributed, then we have for the coarsened errors the well-known formula
(3.6)

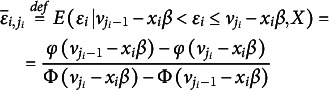



If the errors are not normally distributed but logistically, the formulas for the truncated marginal distribution can be found for example in Johnson et al. (1994) or in Maddala (1983, p. 369). We shall restrict ourselves to the assumption of normally distributed errors.

Since there are no natural units observed, we can only estimate the 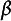 ‘s in (2.1) up to their ratios. A way to make them identifiable is to assume 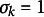 for 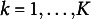 , which is the traditional assumption in Probit and item response analysis.

The sample moment analogue of (3.1) is
(3.7)

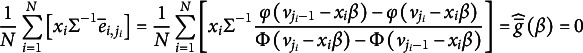

 where 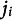 is the index of the interval/block observed for the observation unit 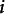 .

Notice that (3.7) is a concise presentation of a system of *K* blocks of *M* equations, each corresponding with one of the elements of the coefficient matrix 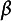 , where we assume that each of the 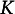 blocks contains 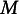 different coefficients 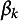 .

The cut-points 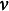 remain to be estimated. There are 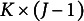 of them. Therefore, we derive another additional set of 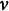 -identifying equations. The cut-points 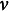 can be easily estimated one by one by applying the following strategy (called binarization). We define for each equation the 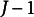 auxiliary binary variables 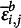 which may assume the lower value 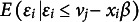 or the upper value 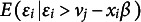 .^5^ We have
(3.8)

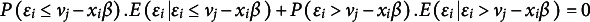



Again, there holds 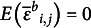 , due to LIE. For the sample counterparts, this implies

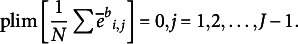



The sample moment analogues are
(3.8a)

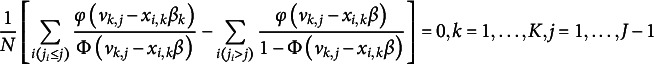



from which the cut-points 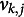 can be easily estimated, as both sums at the left increase in 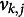 . We notice that these observations are not yet weighted by an error-covariance matrix.

Summing up we are ending with two-equation systems (3.7) and (3.8) from which the parameters 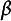 and 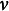 are estimated. This can be done by applying the Generalized Method of Moments (GMM) (Hansen, 1982). We refer to well-known textbooks such as Cameron and Trivedi (2005), Greene (2018) and Verbeek (2017) for elaborate descriptions. The software can be found, e.g., in Stata. We use an iterative calculation scheme. Starting with assuming 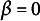 , a first-round yields 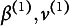 . Taking these values as a starting point we repeat this iterative procedure until convergence, which is rather rapidly reached. The GMM method provides us with an estimate of the covariance matrix of 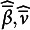 as well, using the well-known “sandwich” formula.

## 4 Polychoric correlations and coefficients of determination

Suppose we have two test items 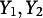 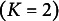 available by which we may, for example, examine an individual or test the effect of a specific therapy or a response to a satisfaction question. For simplicity, we assume both items are yes/no questions. Then we are of course interested to know how correlated the two test items 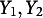 and therewith the responses on the two items are. The latent correlation between items is known in the psychometric literature as the polychoric correlation. The literature on polychoric correlations is massive. We refer out of the host of excellent contributions to the seminal (Olsson, 1979) and the more recent (Liu et al., 2021; Moss and Grønneberg, 2023) for more analysis. The problem is clearly how to estimate correlations between latent variables 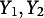 , if we only have a 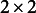 coincidence table at our disposition. We propose the following method.

The latent variables are modeled like (2.1). The latent model is
(4.1)

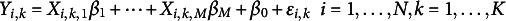

 where in this case 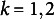 . In this case, we have
(4.2)

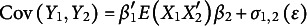

 and more generally for a 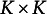 coincidence table we find the 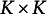 -covariance matrix
(4.3)

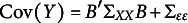

 where 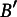 stands for the 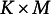 matrix of structural effects and 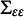 for the latent error covariance matrix. Now, we derive the polychoric correlation from 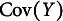 in the usual way, that is, 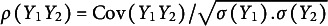 . The covariance matrix 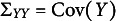 is estimated as
(4.4)

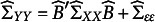

 where 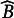 is the estimated matrix of regression coefficients, 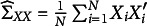 , and 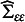 the estimated full error-covariance matrix, as estimated in the Appendix.

We notice that in the case that there are no structural effects found, i.e., 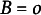 , we still may have non-zero polychoric correlations due to correlated errors. The corresponding correlations are found from the covariance matrix 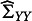 in the usual way.

The matrices 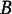 , 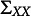 are already consistently estimated. The latent (full) error-covariance matrix 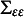 is yet unknown. In the Appendix, we demonstrate how 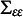 is consistently estimated.

We note that this method does not assume the normality of the random vectors 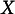 or 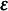 . We may also assume, for example, 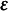 to be logistic. In those cases the formula (3.3) for the generalized residual has to be replaced by the corresponding formula for the logistic, or in fact, any distribution, provided that the covariance matrix is finite.

In the second application below, where we are estimating satisfaction, we present the estimated 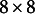 polyserial correlations between satisfactions as the off-diagonal elements in Tables 5–7 and 8a–8e. For the first application in Section 5 we might estimate the polyserial correlations as well but given the panel nature of the data, it is not very interesting.

The relative explanatory power of the equation estimates depends on the question of how volatile the outcomes are due to random errors. Consider (5.1). We have
(4.5)

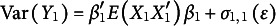



An attractive measure of fit, that is explanatory power is the traditional coefficient of determination
(4.6)

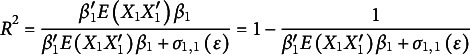



The sample analogue for the first equation is
(4.7)

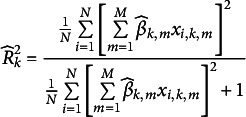

 where 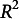 , as postulated in Section 3. This is the same magnitude as proposed by McKelvey and Zavoina (1975). We notice that these numbers may be interpreted as coefficients of determination of the regression equations (5.1) for 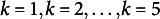 , respectively. Of course, the regression is not performed as 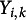 is not observable per individual. However, the regression correlation coefficient can be estimated by a detour using (4.6) and (4.7). We call these satisfaction correlation coefficients. They measure the part of the satisfaction variation, which can be structurally explained by observable traits 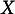 . If there are 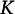 equations, we get 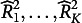 . For curiosity we present in Table 2 our 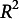 and the McFadden (1974) 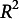 for Ordered Probit side- by -side.

## 5 Two empirical examples and one simulation experiment

In order to evaluate our method empirically we considered two data sets, both part of a 2009–2013 panel data-sequence from the German SOEP-panel data set and a block of eight satisfaction questions in wave 2013 of the SOEP data. The model in Section 5.1 is a set of five time-panel Ordered Probit equations where the errors are correlated. We call this estimation method Feasible Multivariate Ordered Probit (FMOP). It can be generalized to an arbitrary number of panel waves. The second data set consists of eight seemingly unrelated cross-section satisfaction questions, where errors are correlated. It is estimated in Section 5.2. We call this a Seemingly Unrelated Ordered Probit model (SUOP). In addition we present estimations on a simulated data set on request of one of the referees to this paper. The program code may be requested from the first author.

Given the results of the method, it becomes possible to estimate the latent full covariance matrix 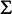 as well. We defer the description of how to estimate the full latent covariance matrix to the Appendix.

### 5.1 Employment status evaluation on a German five-year panel data set

Now we apply the FMOP method to a specific data set. We choose the employment situation of German workers, where we do not pretend to make a study of German employment but merely test the feasibility of the method, using these employment data. Following the lines above, we try to estimate the employment equation and the error covariance matrix using FMOP.

The data are derived from the German, Socio-Economic Panel (SOEP) data set. Households are followed for a period of five successive years (2009–2013). We assume an unstructured error covariance matrix. All explanatory variables are measured as deviations from their averages.

We use the variable employment (variable e11103 in the German SOEP data set) in three self-reported categories “not working,” “part-time working,” and “full-time working.” This implies that the five grids for the years 2009–2013 consist of three intervals each. We assume the explanatory variables “age (18–75 years of age),” “age-squared,” dummy variables for “gender (female = 1),” “marital status: married (reference),” “marital status: single,” “marital status: separated,” “ln(household income minus individual labor income),” “number of children at home,” “years of education,” and dummy variables for “years of education unknown” and “living in East-Germany.” The latent variable is assumed to be generated by the linear equation
(5.1)

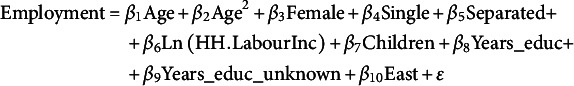



As already said, we assume an “unstructured” 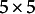 covariance matrix where 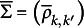 . The results are presented in Table 1. In the left-hand panel, we show the in-between error covariance matrix 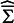 , in the right-hand panel the latent full covariance matrix 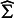 as estimated by the simulation method described in the Appendix. The error correlation over time appears from the right panel to be quite considerable (1.0, 0.8775, 0.7800,…). When we look at the coarsened data the correlation is mitigated by the coarsened observation but still considerable.

**Table 1:** In-between and full error covariance matrices for FMOP.

	In-between covariance matrix (FMOP)					Full covariance matrix (FMOP)				
	2009	2010	2011	2012	2013	2009	2010	2011	2012	2013
2009	0.6303					1.0000				
2010	0.4760	0.6156				0.8775	1.0000			
2011	0.4049	0.4746	0.6149			0.7800	0.8856	1.0000		
2012	0.3695	0.4125	0.4690	0.6131		0.7343	0.7998	0.8837	1.0000	
2013	0.3336	0.3642	0.4059	0.4833	0.6168	0.6867	0.7280	0.7950	0.9079	1.0000

The regression estimates according to FMOP and Ordered Probit (errors independent) are presented in Table 2.

**Table 2:** Regression estimates from FMOP and Ordered Probit.

	Feasible Multivariate			Exact	Ordered Probit (OP)		
	Ordered Probit (FMOP)			observ.			
	Number of obs. = 51760			FMOP	Number of obs. = 51760		
					McF pseudo R^2^ = 0.2523		
					McK-Z R^2^ = 0.5396		
EMPLOYMENT	Coeff.	Std.err.	z	Std.err.	Coeff.	Std.err.	z
AGE	0.3137	0.0059	53.50	0.0038	0.3236	0.0032	102.36
AGE square	−0.0037	0.00006	−58.34	0.00004	−0.0038	0.00003	−114.77
D. FEMALE	−0.7464	0.0223	−33.51	0.0180	−0.7727	0.0115	−67.01
D. SINGLE	0.0778	0.0309	2.52	0.0186	0.0541	0.0202	2.68
sD. SEPARATED	0.0538	0.0250	2.15	0.0164	0.1184	0.0175	6.77
Ln(HHLABOURINC)	0.0071	0.0015	4.65	0.0009	0.0150	0.0013	11.31
# of CHILDREN	−0.2051	0.0114	−17.97	0.0074	−0.2571	0.0077	−33.47
YEARS EDUCATION	0.0789	0.0039	20.03	0.0031	0.0742	0.0021	34.97
D. EDUC. unknown	−0.1172	0.0459	−2.55	0.0318	−0.1985	0.0294	−6.75
D. EAST Germany	−0.0542	0.0237	−2.28	0.0180	−0.0420	0.0128	−3.28
Cut point 1	−0.5892	0.0129	−45.79	–	−0.5984	0.0069	−86.68
Cut point 2	0.3569	0.0123	29.05	–	0.3753	0.0066	5.68

As expected, the regression estimates are of the same order, because both estimators are consistent. The difference is clearly in the calculated standard deviations. All FMOP standard errors are a factor of 1,5 to 2,0 larger than the OP estimates. This is caused because the assumption of error independence by OP instead of the observed strong error correlations is tantamount to a gross exaggeration of the reliability of the data material when we ignore the non-zero error correlations. The difference in standard deviations is a warning signal.

For curiosity we also look at the question of what standard deviations we would have found when we would have had the non-coarsened, that is exact, data material at our disposal. Those standard deviations are estimated by the roots of the diagonal elements of 
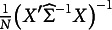
 the elements of which are known. The latent error-covariance matrix 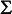 is estimated by 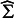 according to the method described in the Appendix. We see from comparison that the FMOP-standard deviations (e.g., for the AGE-coefficient 0.0059) on the basis of the coarsened observations are much larger than the corresponding values found from Ordered Probit theoretical values (0.0032) or for GLS-estimation (0.0038) on the exact latent data. Next to our estimate of we also present the Probit 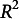 as defined by McFadden (1974). We see that the latter is considerably smaller than ‘our’ 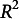 , which follows that of McKenzie-Zavoina (1975).

The computation time in total was 8 seconds. We used a laptop. The computation process can be split up into two parts: the first-stage OP estimation, taking 3 seconds and the second-stage estimation taking another 5 seconds.

We see that employment increases with age until age 42, after which employment decreases (we excluded respondents under 18 years of age and those over 75 years of age). Females are less often employed than males. In households with children, the respondents work less than in childless households. The more additional labor income in the household, the more the respondent works. The more education years one has, the more one works full-time, while respondents from East Germany are less employed than the West Germans.

### 5.2 Seemingly Unrelated Ordered Probit (SUOP) on a block of eight satisfaction questions

**Fig. 1:**
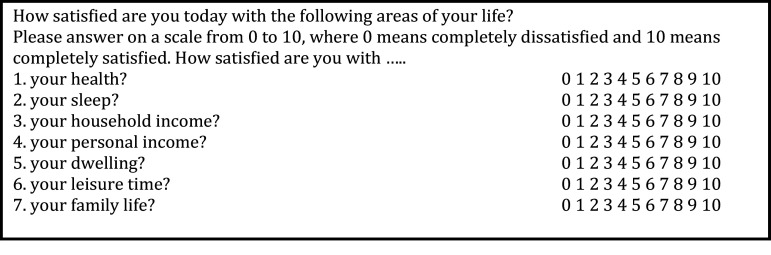
A block of satisfaction questions with respect to various life domains.

In the German panel questionnaire, we find a number of satisfaction questions referring to various life domains, like those presented in Figure 1. Here, we apply the SUOP method.

This type of questioning is abundantly used in marketing research and happiness research. Another very important instance, where the use of SUOP is at hand, is in the analysis of vignettes, also known as factorial surveys in sociological research or as conjoint analysis, now one of the major tools in psychology and marketing research (Green & Srinivasan, 1978; Atzmüller & Steiner 2010; Wallander, 2009; Van Beek et al., 1997).

The data set consists of about 15,000 observation units. Since the original formulation with 11 answer categories made the coarsened observations look very similar to continuous observations, we further coarsened the data into five response categories (0,1,2), (3,4),…,(9,10). In this paper, we apply SUOP analysis to the above-listed block of satisfaction questions with respect to life domains from the 2013 wave of the GSOEP panel. We use the following explanatory variables: age and age-squared, dummies for being female, single and separated, ln(individual labor income), ln(household income *minus* individual labor income), the number of children, the number of years of education, living in East Germany, dummy disability status (0 (no), 1 (yes)), and health rating (1 (bad health),…,5 (very good health)). Our primary objective is to demonstrate the feasibility of SUOP. It stands to reason that for a substantive analysis of domain satisfactions, this model specification is probably too simplistic, however, for our objective, testing the feasibility of SUOP, this specific choice is no problem. In order to avoid that every dependent variable would be explained by the same set of explanatory variables we chose different subsets for each equation.

In Table 3 we present the estimates of the first two equations on Health and Sleep satisfaction. For the full table presenting all eight equation estimates we refer to the Appendix.

**Table 3:** Comparison of the parameter estimates and their s.e.’s for Ordered Probit, Method of Moments, and Maximum Likelihood.

McK-Z *R* ^2^	0.1489		0.1481		0.1154	
*Health Satisfaction*	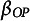	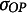	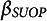	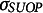	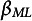	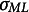
Health: AGE	−0.0695	0.0046	−0.0693	0.0048	−0.0651	0.0045
Health: AGE^2^	0.0006	0.00005	0.0006	0.00005	0.0006	0.00005
Health: D_FEMALE	−0.0182	0.0175	−0.0181	0.0174	−0.0134	0.0166
Health: D_SINGLE	−0.0514	0.0291	−0.0510	0.0293	−0.0570	0.0278
Health: D_SEPARATED	−0.1241	0.0240	−0.1238	0.0243	−0.1108	0.0230
Health: Ln_LABOURINC	0.0289	0.0025	0.0288	0.0026	0.0227	0.0022
Health: CHILDREN	0.0357	0.0123	0.0356	0.0126	0.0308	0.0114
Health: D_EAST	−0.1678	0.0201	−0.1670	0.0196	−0.1402	0.0193
Health: DISABLE	−0.7991	0.0272	−0.7971	0.0268	−0.5825	0.0232
Health: Cut point 1	−1.8277	0.0184	−1.8240	0.0191	−1.8163	0.0181
Health: Cut point 2	−1.1161	0.0131	−1.1094	0.0134	−1.1122	0.0130
Health: Cut point 3	−0.3432	0.0109	−0.3415	0.0109	−0.3399	0.0107
Health: Cut point 4	0.9468	0.0123	0.9442	0.0124	0.9409	0.0122
McK-Z *R* ^2^	0.0278		0.0278		0.0196	
*Sleep Satisfaction*	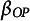	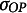	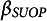	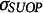	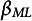	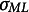
Sleep: AGE	−0.0380	0.0041	−0.0381	0.0042	−0.0348	0.0041
Sleep: AGE^2^	0.0004	0.00004	0.0004	0.00004	0.0003	0.00004
Sleep: D. FEMALE	−0.1213	0.0172	−0.1213	0.0172	−0.1204	0.0164
Sleep: D. SINGLE	0.0508	0.0302	0.0505	0.0307	0.0594	0.0284
Sleep: D. SEPARATED	−0.0762	0.0253	−0.0762	0.0260	−0.0658	0.0238
Sleep: Ln(HH.LABOURINC)	0.0067	0.0021	0.0066	0.0020	0.0062	0.0018
Sleep: # of CHILDREN	0.0219	0.0121	0.0220	0.0122	0.0164	0.0113
Sleep: YEARS of EDUCATION	0.0316	0.0032	0.0315	0.0031	0.0117	0.0028
Sleep: D. EAST GERMANY	−0.1063	0.0199	−0.1065	0.0196	−0.0763	0.0192
Sleep: Cut point 1	−1.6918	0.0175	−1.6912	0.0174	−1.7036	0.0174
Sleep: Cut point 2	−0.9791	0.0123	−0.9800	0.0123	−0.9831	0.0123
Sleep: Cut point 3	−0.9251	0.0106	−0.2970	0.0106	−0.2940	0.0104
Sleep: Cut point 4	0.7286	0.0114	0.7269	0.0115	0.7150	0.0113

In the first two columns we present the initial Probit estimates and their s.e.’s. In columns 3, 4 we present the corresponding SUOP-estimates and their s.e’s. The two right-hand columns 5, 6 give the corresponding estimates by means of the ML-method. We take the cmp results as the touchstone of our comparison.

Our first conclusion is that the three methods OP, SUOP, ML yield estimates which do not differ significantly in most cases. This is not surprising as the three estimators are consistent. The standard deviations of the SUOP-estimators seem to be slightly larger than the ML-estimators, but the differences are mostly negligible.

In Table 4 we present the full correlation matrices as estimated by SUOP (estimation according to Appendix) and ML (according to Stata), respectively.

**Table 4:** Full error correlation matrices compared for SUOP and ML.

Residual								Stand.
Corr. SUOP	Health	Sleep	HH inc.	Ind inc.	Dwelling	Leisure	Family life	living
Health	1.0000							
Sleep	0.5328	1.0000						
HH. inc.	0.3196	0.2758	1.0000					
Ind. inc.	0.2851	0.2367	0.8414	1.0000				
Dwelling	0.3103	0.2894	0.4521	0.3769	1.0000			
Leisure	0.3246	0.3359	0.3274	0.2812	0.4557	1.0000		
Family life	0.3585	0.3295	0.3327	0.2838	0.4526	0.4743	1.0000	
Stand. living	0.3964	0.2520	0.7173	0.6044	0.5551	0.4626	0.5930	1.0000
Residual Corr. ML	Health	Sleep	HH inc.	Ind inc.	Dwell ing	Leisure	Family life	Stand. living
Health	1.0000							
Sleep	0.5487	1.0000						
HH. inc.	0.3280	0.2802	1.0000					
Ind. inc.	0.2919	0.2487	0.8266	1.0000				
Dwelling	0.3144	0.2990	0.4530	0.3869	1.0000			
Leisure	0.3301	0.3463	0.3323	0.2899	0.4635	1.0000		
Family life	0.3643	0.3405	0.3355	0.2849	0.4563	0.4800	1.0000	
Stand. living	0.3973	0.3492	0.7099	0.6118	0.5582	0.4610	0.5930	1.0000

We see that of the 75 SUOP-estimated regression coefficients 17 fall out of the ML-confidence intervals. For the estimates of the correlation matrix we find a similar result.^6^ Three of the 28 SUOP estimated correlation coefficients are just outside the ML-confidence intervals.

The polychoric correlation matrix is presented in Table 5.

**Table 5:** The polychoric correlation matrix.

Residual								Stand.
Corr. SUOP	Health	Sleep	HH inc.	Ind inc.	Dwelling	Leisure	Family life	living
Health	1.0000							
Sleep	0.7372	1.0000						
HH. inc.	0.7626	0.6347	1.0000					
Ind. inc.	0.7389	0.6028	0.9418	1.0000				
Dwelling	0.6985	0.5998	0.7752	0.7338	1.0000			
Leisure	0.6962	0.6258	0.7111	0.6704	0.7287	1.0000		
Family life	0.7496	0.6372	0.7550	0.7101	0.7558	0.7521	1.0000	
Stand. living	0.8015	0.6728	0.9220	0.8705	0.8169	0.7680	0.8538	1.0000

A naïve approach is to assign the values 0,1,…,9,10 to the satisfaction values and to calculate the Pearson correlations on that basis. This assignment is conform to daily usage, where average satisfaction values in a sample are also based on this assignment practice.

The Pearson correlations are presented in Table 6. We see that there is a considerable difference between Tables 5 and 6. We prefer 5 to 6, as the cardinalization by 0,1..,10 is arbitrary and might be replaced by another one (0,2,3,…) yielding a different Table 6, while the polyserial correlations are based on endogenous cardinalization. We notice that all Pearson correlations in Table 6 are considerably smaller than the corresponding numbers in Table 5.

**Table 6:** The Pearson correlation matrix. (scale 0–10).

Pearsoncorr.								Stand.
scale (0–10)	Health	Sleep	HH inc.	Ind inc.	Dwelling	Leisure	Family life	living
Health	1.0000							
Sleep	0.5185	1.0000						
HH. inc.	0.3331	0.2829	1.0000					
Ind. inc.	0.2899	0.2575	0.7761	1.0000				
Dwelling	0.2414	0.2635	0.4307	0.3580	1.0000			
Leisure	0.2121	0.2804	0.2921	0.2448	0.4047	1.0000		
Family life	0.3027	0.2965	0.3331	0.2599	0.4085	0.3985	1.0000	
Stand. living	0.3852	0.3325	0.7112	0.5872	0.5201	0.3806	0.5387	1.0000

The computation process can be split up into three parts: the first-stage OP estimation took 1.8 seconds on our ASUS VivoBook 15 laptop, and the second-stage SUOP estimation took another 111 seconds. The whole calculation requires less than two minutes. The ML estimations by cmp (default) took 7 hours. In the SUOP method, we use the in-between covariance matrix 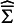 and not the full covariance estimate. The computation time depends on the sample size 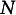 , the size 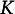 of the error covariance matrix, and the capacity of our laptop. In this example 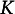 = 8. We see that for the ML method, the time increases non-linearly with 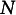 . The number 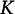 seems to be important as well. For 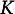 = 2 equations both methods are roughly equally fast, where SUOP takes 11 seconds and ML-cmp 10.5 seconds for 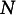 = 15535. For 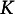 = 3 the SUOP computation increases to 16 seconds, while the ML method requires already 1,241 seconds. This is caused, it seems, by the fact that ML has to evaluate a lot of 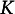 *-*dimensional integrals. A colleague of ours (an expert Stata user) observed, quoting the “options” in the Stata text, that cmp uses the GHK-simulation method for evaluating the needed integrals and that in the default option cmp uses 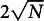 draws per evaluated likelihood. In the present case, this is about 250 simulations per observation. Cappellari and Jenkins (2003) suggested that for a large number of observations, the number of draws can be considerably reduced without severe efficiency loss. According to our colleague by taking 5 draws per likelihood we would reduce the computation time from the reported 7 hours to 8 minutes with only a slight efficiency reduction. That is probably still significantly slower than the new method, but the revision would be material. We followed this suggestion and found indeed comparable estimates for the coefficients 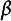 . To our surprise the standard deviations for the five draws were not significantly different from the 250 draws version. This seems to indicate that in the assessment of variance the additional contribution caused by the simulation variance is not taken into account.

Clearly, if we would reduce the number of equations from eight to a more manageable four or two equations and/or reduce the number of observations, both the ML and the SUOP methods would perform much faster.

Our conclusion is that the SUOP method is faster than the ML method. We are unable to say whether the Stata procedure cmp is to blame and could be improved or whether this is a general feature of the ML-GHK procedure. It might also be that we could have reduced the ML computation time by choosing specific options instead of the default procedure. Choosing too severe tolerance levels for the iterations involved would have increased the computation time in exchange for more exact confidence intervals. However, given that the cmp outcomes have about the same confidence intervals as our SUOP outcomes we do not believe that the tolerance levels chosen in cmp were more severe than in our method (see Table 8f).

### 5.3 A simulated example

Finally, we apply the estimation method to a simulated data set. We simulated a hard data set of 10,000 observations. We generated the set as follows. We assumed a latent model

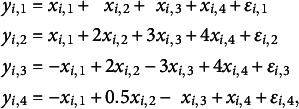

 where, 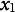 is normal 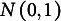 , where 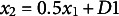 with *D*1 a dummy variable equal to +1 or −1 with 50% each, where 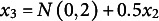 , and where 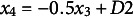 and *D*2 is drawn to equal +1, 0, or −1 with a chance of 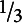 each.

The error vector 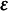 is i.i.d. 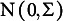 with

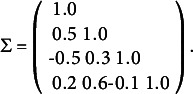



We notice that all four variables 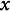 and the error vector has an expectation equal to zero. In order to avoid that (the non-conditioned) 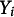 is approximately normal, we restricted the explanatory variables to a small number of four and we chose those variables to be non-normal and correlated, such that the structural part 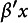 does not tend to normality. Our first aim is to look for the distribution of the exact data. The expectation 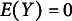 , the empirical mean equals 0.0160 and the variance var(*Y*) equals 2.603. The correlation matrix of the variables 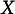 , and 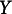 is the 8 × 8 matrix in Table 1.

Cut points are defined as 
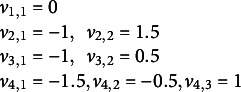


We define the response indicators: 
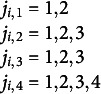


The model is iteratively estimated by the FMOP method. 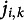 is the interval index by respondent *i* for equation *k*, corresponding with the four equations *k* = 1,…,4. We start with iteration *t* = 0 for 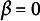 . We define the under- and upper residuals

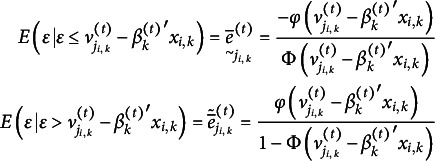



We define the sets of respondents 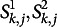 (*k* = 1,…4; *j* = 1,…, 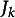 ) who are in the response categories 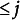 or 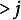 respectively. We solve the equations
(5c.1)

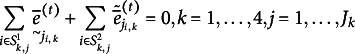

 for 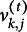 and find estimated cut-points 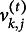 in the *t* ^th^ iteration. These cut-points 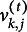 are substituted to define the generalized residuals. The estimated generalized residuals 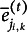 in the 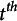 iteration are

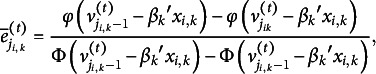

 where 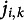 is the interval index by respondent *i* for equation *k.* corresponding with the four equations *k* = 1,…,4. We now define the 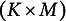 orthogonality conditions



(5c.2)

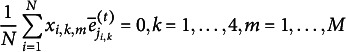



We have now two equation systems (5c.1) and (5c.2), which are simultaneously solved. Then we calculate the in-between error covariance matrix

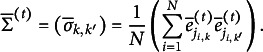



We repeat (5c.1) and (5c.2) with the new 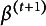 and 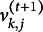 , and find new estimates. We repeat (5c.1) and (5c.2) after weighting with the inverse covariance matrix solving




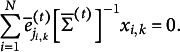



In the end we estimate the corresponding covariance matrix of the estimators 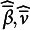 by the well-known sandwich formula.

We estimate each non-diagonal element 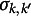 of the latent full covariance matrix from the corresponding element 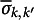 of the in-between error covariance matrix. The method is described in detail in the Appendix.

We conclude that the method is stable in the number *N of observations* and it does not differ significantly from the cmp-estimates.

**Table 7:** Beta’s, Standard errors and Error correlations (*N =* 10,000).

N = 10000	beta’s MoM	s.e. MoM	beta’s cmp	s.e. cmp
Eq.y1: var. x1	0.9794	0.0264	0.9799	0.0269
Eq.y1: var. x2	0.9910	0.0240	0.9927	0.0241
Eq.y1: var. x3	0.9715	0.0222	0.9715	0.0223
Eq.y1: var. x4	0.9694	0.0281	0.9703	0.0282
Eq.y1: cutp. 1	−0.0024	0.0193	−0.0072	0.0191
Eq.y2: var. x1	1.0329	0.0290	1.0557	0.0279
Eq.y2: var. x2	1.9859	0.0396	1.9991	0.0387
Eq.y2: var. x3	3.0286	0.0550	3.0558	0.0535
Eq.y2: var. x4	4.0158	0.0733	4.0526	0.0711
Eq.y2: cutp. 1	−1.0215	0.0321	−1.0154	0.0311
Eq.y2: cutp. 2	1.4972	0.0386	1.5026	0.0358
Eq.y3: var. x1	−1.0234	0.0444	−1.0065	0.0406
Eq.y3: var. x2	2.0183	0.0640	1.9981	0.0586
Eq.y3: var. x3	−3.0813	0.0847	−3.0441	0.0805
Eq.y3: var. x4	4.1387	0.1152	4.0610	0.1097
Eq.y3: cutp. 1	−0.9753	0.0447	−0.9675	0.0428
Eq.y3: cutp. 2	0.5115	0.0407	0.4993	0.0387
Eq.y4: var. x1	−1.0079	0.0212	−1.0078	0.0209
Eq.y4: var. x2	0.4957	0.0169	0.4931	0.0165
Eq.y4: var. x3	−1.0180	0.0173	−1.0143	0.0171
Eq.y4: var. x4	0.9727	0.0224	0.9696	0.0218
Eq.y4: cutp. 1	−1.5233	0.0282	−1.5198	0.0273
Eq.y4: cutp. 2	−0.5125	0.0233	−0.5165	0.0223
Eq.y4: cutp. 3	1.0335	0.0253	1.0289	0.0245

Estimated Full correlation matrix MoM				Estimated Full correlation matrix cmp			
1.0000				1.0000			
0.4496	1.0000			0.4703	1.0000		
−0.5450	0.2527	1.0000		−0.4841	0.2306	1.0000	
0.1934	0.5877	−0.0846	1.0000	0.2110	0.6266	−0.0857	1.0000

**Table 8a:** Beta’s, Standard errors and Error correlations (*N =* 5,000) (Base dataset of 10000 cases, only every second case is used).

N = 5000	beta’s MoM	s.e. MoM	beta’s cmp	s.e. cmp
Eq.y1: var. x1	1.0192	0.0385	1.0161	0.0392
Eq.y1: var. x2	0.9952	0.0341	0.9949	0.0344
Eq.y1: var. x3	0.9598	0.0319	0.9573	0.0312
Eq.y1: var. x4	0.9484	0.0398	0.9460	0.0397
Eq.y1: cutp. 1	−0.0330	0.0273	−0.0361	0.0270
Eq.y2: var. x1	1.0832	0.0418	1.0969	0.0402
Eq.y2: var. x2	1.9317	0.0552	1.9383	0.0534
Eq.y2: var. x3	3.0125	0.0790	3.0381	0.0749
Eq.y2: var. x4	4.0011	0.1053	4.0333	0.0997
Eq.y2: cutp. 1	−1.0462	0.0462	−1.0508	0.0442
Eq.y2: cutp. 2	1.4679	0.0542	1.4743	0.0501
Eq.y3: var. x1	−1.0418	0.0636	−1.0452	0.0567
Eq.y3: var. x2	1.9988	0.0847	2.0070	0.0807
Eq.y3: var. x3	−3.0718	0.1113	−3.0620	0.1128
Eq.y3: var. x4	4.1625	0.1512	4.1297	0.1548
Eq.y3: cutp. 1	−0.9182	0.0619	−0.8988	0.0598
Eq.y3: cutp. 2	0.6152	0.0562	0.6031	0.0554
Eq.y4: var. x1	−0.9814	0.0300	−0.9829	0.0292
Eq.y4: var. x2	0.4882	0.0238	0.4860	0.0233
Eq.y4: var. x3	−1.0281	0.0246	−1.0261	0.0244
Eq.y4: var. x4	0.9506	0.0322	0.9492	0.0306
Eq.y4: cutp. 1	−1.5350	0.0398	−1.5408	0.0390
Eq.y4: cutp. 2	−0.5381	0.0327	−0.5369	0.0321
Eq.y4: cutp. 3	1.0050	0.0362	1.0132	0.0343

Estimated Full correlation matrix MoM				Estimated Full correlation matrix cmp			
1.0000				1.0000			
0.5288	1.0000			0.4930	1.0000		
−0.3185	0.3089	1.0000		−0.3905	0.3245	1.0000	
0.3014	0.6468	−0.1165	1.0000	0.2201	0.6138	−0.0981	1.0000

0.3014: 95% confidence interval cmp [0.1462: 0.2916]

**Table 8b:** Beta’s, Standard errors and Error correlations (*N =* 2,000) (Base dataset of 10000 cases, only every fifth case is used).

N = 2000	beta’s MoM	s.e. MoM	beta’s cmp	s.e. cmp
Eq.y1: var. x1	0.9929	0.0574	0.9814	0.0590
Eq.y1: var. x2	0.9259	0.0507	0.9186	0.0509
Eq.y1: var. x3	0.9448	0.0512	0.9392	0.0483
Eq.y1: var. x4	0.9605	0.0628	0.9593	0.0609
Eq.y1: cutp. 1	−0.0165	0.0418	−0.0081	0.0414
Eq.y2: var. x1	0.9729	0.0643	0.9747	0.0588
Eq.y2: var. x2	1.9411	0.0822	1.9198	0.0827
Eq.y2: var. x3	2.9799	0.1168	2.9654	0.1138
Eq.y2: var. x4	3.9235	0.1548	3.9077	0.1502
Eq.y2: cutp. 1	−1.0068	0.0705	−0.9718	0.0677
Eq.y2: cutp. 2	1.4167	0.0828	1.4069	0.0746
Eq.y3: var. x1	−1.0846	0.0992	−1.0725	0.0990
Eq.y3: var. x2	2.1905	0.1320	2.2170	0.1474
Eq.y3: var. x3	−3.3765	0.1985	−3.4092	0.2109
Eq.y3: var. x4	4.4998	0.2566	4.5139	0.2813
Eq.y3: cutp. 1	−1.2270	0.1100	−1.2034	0.1120
Eq.y3: cutp. 2	0.5252	0.0976	0.5239	0.0939
Eq.y4: var. x1	−0.9667	0.0465	−0.9641	0.0458
Eq.y4: var. x2	0.4765	0.0372	0.4680	0.0362
Eq.y4: var. x3	−1.0109	0.0378	−0.9994	0.0386
Eq.y4: var. x4	0.9413	0.0511	0.9278	0.0472
Eq.y4: cutp. 1	−1.5391	0.0629	−1.5537	0.0605
Eq.y4: cutp. 2	−0.5099	0.0509	−0.5206	0.0493
Eq.y4: cutp. 3	0.9616	0.0541	0.9675	0.0527

Estimated Full correlation matrix MoM				Estimated Full correlation matrix cmp			
1.0000				1.0000			
0.5982	1.0000			0.5334	1.0000		
−0.2635	0.3972	1.0000		−0.3878	0.2402	1.0000	
0.3114	0.6242	0.0889	1.0000	0.2159	0.6098	−0.0263	1.0000

**Table 8c:** Beta’s, Standard errors and Error correlations (*N =* 1,000) (Base dataset of 10000 cases, only every tenth case is used).

N = 1000	beta’s MoM	s.e. MoM	beta’s cmp	s.e. cmp
Eq.y1: var. x1	0.9058	0.0798	0.8950	0.0820
Eq.y1: var. x2	0.9667	0.0719	0.9531	0.0718
Eq.y1: var. x3	0.8916	0.0726	0.8826	0.0651
Eq.y1: var. x4	0.9127	0.0880	0.9065	0.0839
Eq.y1: cutp. 1	0.0109	0.0581	0.0193	0.0578
Eq.y2: var. x1	1.0706	0.0998	1.0462	0.0885
Eq.y2: var. x2	1.8804	0.1089	1.8831	0.1162
Eq.y2: var. x3	2.9807	0.1617	2.9809	0.1636
Eq.y2: var. x4	3.9341	0.2122	3.9386	0.2157
Eq.y2: cutp. 1	−1.0250	0.0946	−1.0196	0.0955
Eq.y2: cutp. 2	1.3482	0.1172	1.3420	0.1055
Eq.y3: var. x1	−1.1157	0.1235	−1.1258	0.1419
Eq.y3: var. x2	2.1102	0.1724	2.1565	0.1987
Eq.y3: var. x3	−3.2868	0.2639	−3.3482	0.2912
Eq.y3: var. x4	4.4391	0.3299	4.5149	0.3847
Eq.y3: cutp. 1	−1.1548	0.1573	−1.1448	0.1512
Eq.y3: cutp. 2	0.5539	0.1232	0.5435	0.1322
Eq.y4: var. x1	−1.0006	0.0658	−1.0014	0.0664
Eq.y4: var. x2	0.4593	0.0526	0.4538	0.0507
Eq.y4: var. x3	−1.0533	0.0529	−1.0494	0.0562
Eq.y4: var. x4	0.9190	0.0742	0.9095	0.0665
Eq.y4: cutp. 1	−1.6660	0.0938	−1.7127	0.0892
Eq.y4: cutp. 2	−0.5368	0.0729	−0.5796	0.0724
Eq.y4: cutp. 3	1.0163	0.0762	1.0410	0.0778

Estimated Full correlation matrix MoM				Estimated Full correlation matrix cmp			
1.0000				1.0000			
0.7347	1.0000			0.5360	1.0000		
−0.3041	0.0570	1.0000		−0.3291	0.2410	1.0000	
0.1500	0.6412	−0.1904	1.0000	0.2013	0.5261	−0.1288	1.0000

0.7347: 95% confidence interval cmp [0.3862: 0.6583]

**Table 8d:** Beta’s, Standard errors and Error correlations (*N =* 1,000) (A newly created dataset of 1000 cases).

N = 1000	beta’s MoM	s.e. MoM	beta’s cmp	s.e. cmp
Eq.y1: var. x1	0.9490	0.0846	0.9536	0.0827
Eq.y1: var. x2	0.9997	0.0726	1.0142	0.0747
Eq.y1: var. x3	0.9368	0.0702	0.9446	0.0696
Eq.y1: var. x4	0.9916	0.0871	0.9944	0.0908
Eq.y1: cutp. 1	0.0282	0.0604	0.0380	0.0599
Eq.y2: var. x1	0.8773	0.0956	0.8964	0.0795
Eq.y2: var. x2	1.9966	0.1537	1.9868	0.1219
Eq.y2: var. x3	2.9308	0.2170	2.9092	0.1597
Eq.y2: var. x4	3.8298	0.2892	3.8065	0.2112
Eq.y2: cutp. 1	−0.9652	0.0989	−0.9571	0.0952
Eq.y2: cutp. 2	1.2944	0.1303	1.3508	0.1049
Eq.y3: var. x1	−1.0870	0.1472	−1.0583	0.1484
Eq.y3: var. x2	2.1040	0.2332	2.1304	0.2109
Eq.y3: var. x3	−3.2458	0.2952	−3.2709	0.2838
Eq.y3: var. x4	4.2512	0.4029	4.3088	0.3815
Eq.y3: cutp. 1	−1.0302	0.1601	−1.0904	0.1513
Eq.y3: cutp. 2	0.6065	0.1224	0.5906	0.1338
Eq.y4: var. x1	−0.9638	0.0650	−0.9645	0.0649
Eq.y4: var. x2	0.4585	0.0521	0.4732	0.0518
Eq.y4: var. x3	−0.8987	0.0531	−0.9015	0.0500
Eq.y4: var. x4	0.9384	0.0712	0.9204	0.0672
Eq.y4: cutp. 1	−1.4835	0.0854	−1.4945	0.0842
Eq.y4: cutp. 2	−0.5339	0.0690	−0.5255	0.0680
Eq.y4: cutp. 3	0.8221	0.0821	0.8277	0.0699

Estimated Full correlation matrix MoM				Estimated Full correlation matrix cmp			
1.0000				1.0000			
0.5582	1.0000			0.3511	1.0000		
−0.5354	0.4227	1.0000		−0.5507	0.1285	1.0000	
0.3158	0.7640	−0.3565	1.0000	0.1896	0.6688	−0.1589	1.0000

0.5582: 95% confidence interval cmp [0.1834: 0.4989] 0.4227: 95% confidence interval cmp [−0.1869: 0.4199]

**Table 8e:** Beta’s, Standard errors and Error correlations (*N =* 1,000) (Again a newly created dataset of 1000 cases).

	beta’s MoM	s.e. MoM	beta’s cmp	s.e. cmp
Eq.y1: var. x1	0.9592	0.0809	0.9417	0.0800
Eq.y1: var. x2	0.9477	0.0738	0.9352	0.0708
Eq.y1: var. x3	0.9064	0.0648	0.8982	0.0652
Eq.y1: var. x4	0.9397	0.0831	0.9374	0.0833
Eq.y1: cutp. 1	0.0766	0.0588	0.0701	0.0577
Eq.y2: var. x1	0.9359	0.0837	0.9287	0.0860
Eq.y2: var. x2	2.0244	0.1159	2.0294	0.1236
Eq.y2: var. x3	2.8800	0.1441	2.8982	0.1618
Eq.y2: var. x4	3.8478	0.1961	3.8657	0.2161
Eq.y2: cutp. 1	−0.9785	0.0996	−0.9942	0.0962
Eq.y2: cutp. 2	1.4898	0.1128	1.4802	0.1161
Eq.y3: var. x1	−0.8380	0.1291	−0.8675	0.1328
Eq.y3: var. x2	1.8184	0.1703	1.8429	0.1721
Eq.y3: var. x3	−2.8861	0.2167	−2.8964	0.2404
Eq.y3: var. x4	3.8474	0.2721	3.8491	0.3136
Eq.y3: cutp. 1	−1.0368	0.1240	−1.0327	0.1390
Eq.y3: cutp. 2	0.3357	0.1239	0.3267	0.1197
Eq.y4: var. x1	−1.0838	0.0679	−1.0809	0.0705
Eq.y4: var. x2	0.5400	0.0531	0.5334	0.0530
Eq.y4: var. x3	−1.0304	0.0550	−1.0243	0.0558
Eq.y4: var. x4	1.0777	0.0685	1.0711	0.0698
Eq.y4: cutp. 1	−1.5268	0.0823	−1.5160	0.0856
Eq.y4: cutp. 2	−0.4549	0.0726	−0.4652	0.0695
Eq.y4: cutp. 3	1.0477	0.0764	1.0275	0.0767

Estimated Full correlation matrix MoM				Estimated Full correlation matrix cmp			
1.0000				1.0000			
0.6303	1.0000			0.6316	1.0000		
−0.1647	0.5655	1.0000		−0.1230	0.2274	1.0000	
0.1451	0.6184	0.0078	1.0000	0.1891	0.5982	0.0324	1.0000

(0.5655: 95% confidence interval cmp [−0.1068: 0.5155)

**Table 8f:** The correlation matrix of the variables (*N* = 10,000).

	x1	x2	x3	x4	y1	y2	y3	y4
x1	1.0000							
x2	0.8797	1.0000						
x3	−0.3812	−0.2516	1.0000					
x4	−0.4803	−0.3452	0.9240	1.0000				
y1	0.6278	0.4450	−0.1164	−0.3281	1.0000			
y2	0.7033	0.6220	−0.0943	−0.2136	0.4566	1.0000		
y3	0.5598	0.5484	−0.9312	−0.8929	0.1250	0.2659	1.0000	
y4	−0.2500	−0.0479	0.9196	0.8447	−0.0921	−0.2116	−0.7871	1.0000

## 6 Concluding remarks

In this paper, we suggest a new approach to the statistical analysis of ordinal data, where the errors are supposed to be correlated. The basic idea is that the ordinally observed dependent variables reflect latent continuously-valued random variables 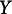 and that the observations are coarsened corresponding to an interval grid 
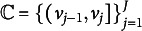
 of 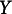 on the real axis, where the unknown cut-points 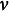 have to be estimated as well. Each observed category corresponds to one interval on the real axis. For cases where 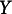 is more dimensional, say *K*, and errors are correlated there is mostly a formidable impediment. The usual ML-estimation procedure requires to evaluate likelihoods, which are multivariate normal integrals over 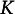 -dimensional blocks. If this has to be performed this is very cumbersome and time -consuming. In this paper we show that estimation of the latent generating model behind the coarsened observations of dependent variables can be done in a much simpler way than usual without the need for multi-dimensional integration or large-scale simulations.

In our approach, we depart from the requirement that the difference between the observation 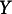 and its predictor 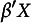 , that is 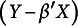 , cannot be further explained by 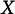 . This is translated into the zero-covariance conditions (2.5) and gives those conditions a significance on their own. When the errors are normally distributed this coincides with the ML-conditions.

The identifying moment conditions are found by substituting the residuals in the regular zero covariance-conditions for exact data by the corresponding generalized residuals corresponding to the ordinal data.

The approach closely resembles the traditional GLS- and SUR-approaches used to estimate linear models on exactly observed dependent variables.

For this method, an assumption about the marginal distributions of the error vector is required. We choose for normality, which enables us to use (3.6). Although we restricted ourselves to assuming normal errors, it is not difficult to generalize this method for other error distributions as well, where the logistic and the lognormal are the foremost candidates (see in those cases, e.g., Maddala (1983, p. 369) for the formulae of the generalized residuals). The estimation method remains unchanged.

This approach seems to smoothly close the gap between the analysis of exactly observable data and qualitative ordinal data. We saw in the above examples that the effect on variances (confidence bands and intervals) caused by SUOP compared to OP is in some cases small and in other cases large. The regression coefficients are mostly similar, which is not surprising as both estimators are consistent estimators. This is also the case for the comparison between traditional OLS and SUR estimates in traditional econometrics. The advantage lies in the possibility to account for error correlations, caused by using the additional information supplied by the error correlations. Standard error deviations are assessed without assuming a specific structure of the covariance matrix before estimation. In the panel data example in Section 5.1 it appears that the standard deviations of the estimates are doubled or more taking error correlation into account. Hence, in this case the reliability reduction when taking error correlations into account is huge.

In this paper, we focus on the qualitative versions of FMOP and SUOP of FGLS and SUR. However, this method seems generally appropriate for two broad types of model estimation situations characterized by Roodman (2011) as:

“1) those in which a truly recursive data-generating process is posited and fully modeled, and

2) those in which there is simultaneity but instruments allow the construction of a recursive set of equations, as in two-stage least squares (2SLS).”

Our method may be compared with the methods, (based on the GHK algorithm), developed by Cappellari and Jenkins (2003), based on simulated moments (Hajivassiliou & McFadden, 1998; Roodman, 2007, 2020). Those methods aim at getting numerical estimates of the log-likelihood by simulation and finding, by variation of the unknown parameters to be estimated, which parameter values maximize the simulated log-likelihood of the sample. This requires the repeated evaluation of multiple normal integrals and makes the procedure time-consuming. In our approach, we do not need to evaluate multiple integrals or large-scale simulations, and therefore the method is not restricted with respect to the size 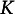 of the equation system. Moreover, we can handle an arbitrary number 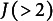 of outcome categories. We do not have to restrict ourselves to dichotomous (biprobit) data only. The method can be used for any number of equations 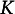 and any number of interval categories 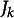 . For instance, in our SUOP-example (Section 5.2) we estimated eight equations, 75 effects and 32 cut points simultaneously. It is obvious that direct observation is a limiting case of coarsening and consequently the methos may also be used when the data set consists of a mixture of directly observed and ordinal data. Classical least-squares based estimation methods on exactly observed data may be seen as a specific limiting case.

Our estimation method appears to require only a few minutes of computing time, which compares favorably with the traditional methods each of which requires much more time. The method may be interpreted as a generalization of classical least squares models that deal with exact observations to include the estimation of models on the basis of more-dimensional ordered probit-type observations.

In this paper, we restricted ourselves to the most straightforward OP observation mode. In a forthcoming study, we will generalize this approach to tackle the case where the sample consists of a mixture of categorical, censored, and exactly observed data.

## Figures and Tables

**Figure. A1:**
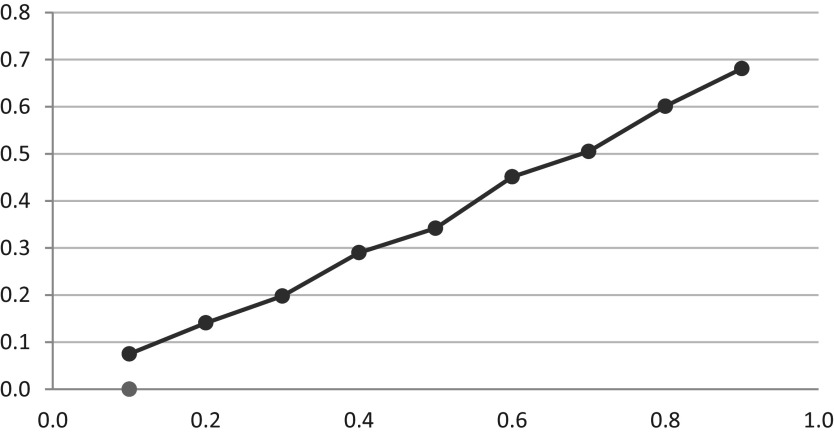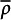 as a function of 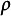 .

## Footnotes

1
Capitals will be used for random variables, vectors, and matrices. We denote the zero-vector by o = (0,…,0). Disturbance terms will be in Greek letters. Roman letters will be used for constants and realizations of random variables. Matrices will be denoted by capitals as well to conform to the traditional regression formulas. Indexes will be suppressed where the interpretation will be clear from the context. The ML-estimator of a parameter vector  is denoted by  . The corresponding estimator from coarsened data is denoted by  . An overbar above a symbol denotes a sample average.

2
The original least squares method is due to Gauss (1809) and Legendre (1805). “Sur la Méthode des moindres quarrés”. See, also, Stigler (1981) for a historical survey.

3
The important issue of whether the coarsened sample data  contain sufficient information to identify estimators of  and the unknown cut-points in  is considered in Greene and Hensher (2010) among others.

4
Notice that  .

5
This trick, called “binarization” is suggested by, e.g., Chris Muris, 2017. “Estimation in the Fixed-Effects Ordered Logit Model,” *The Review of Economics and Statistics*, vol. 99(3), pages 465–477, July. The term has been used before in computer science.

6
The cmp-procedure provides confidence intervals for the correlation estimates.

## Appendix

### A Estimation of the latent error covariance matrix Σ

For the estimation of the latent covariance matrix starting from the estimated  -estimates and the in-between covariance matrix  we propose the following method. We make use of the fact that in order to estimate the true error covariances  by  for two OP-equations we notice that for each pair  we only have to look for the bivariate marginal distribution of  . Consider for instance a cluster of three observations (1, 2, 3). The sample likelihood is  , where  and  and  similarly defined. However, the bivariate marginal likelihood provides the same information on  as the trivariate full likelihood. In the  -dimensional covariance matrix there are  different non-diagonal elements. For instance, if  = 8 as in the model in Section 6, this boils down to 28 simple two-dimensional estimations.

Applying the Gram-Schmidt decomposition, there holds for the pair  .

(A.1)

where  are independent  drawings.^7^ The coefficient  guarantees that  . We have  . When we consider the estimated in-between error covariance  , then its value depends only on the two one-dimensional grids ℂ*_ik_* and ℂ*_i,k’_* and  . We may now simulate  for various values of  by means of (A.1), and calculate the corresponding in-between covariances  , corresponding to the grids ℂ*_ik_* and ℂ*_i,k’_*. We notice that the dependent variable vector  is observed according to a uniform  -independent gridℂ*_k_*  , while the errors  are observed according to  -dependent individual grids

We consider the set of all two-dimensional grids for all observation units. {ℂ*_ik_*, ℂ*_i,k’_*}  = ℂ*_k,k’_*

We write for the sample in-between covariance

We estimate the value of the latent  by comparing the observed sample in-between covariance  with its simulated counterpart  for various values of the latent  . It appears that there is one value  solving  . In order to gain insight into the relationship between the latent  and the corresponding in-between covariance  we did some simulation experiments for three different two-dimensional grids. In Table A1 we present three different two-dimensional grids with different  values for different values of  . We describe one example in detail. Consider the two-dimensional grid with ℂ*_1_*  , ℂ*_2_*  in the middle part of Table A1. We simulate a sample from a two-dimensional normal distribution with a correlation with  = 0.1. We find an in-between covariance  = 0.043. For  = 0.2 we find a corresponding value of  = 0.087, and so on. In Table A1 we present the relationship between  and  for three different two-dimensional grids. We found that the function  =  is monotonically increasing in  for all grids we tested. See also Aitkin(1964). We conclude that for a given grid (ℂ*_1_*,ℂ*_2_*) the function  =  is monotonically increasing in  .

**Table A1:** Relation between covariance  and in-between covariance  .

grid			grid			grid		
ν_11_ = −0.5	0.1	0.075	ν_1_ = 0.0	0.1	0.043	ν_11_ = −1.0	0.1	0.032
ν_12_ = 0.0	0.2	0.141		0.2	0.087	ν_12_ = 2.0	0.2	0.051
ν_13_ = 0.75	0.3	0.198		0.3	0.128		0.3	0.092
	0.4	0.290	ν_2_ = 0.0	0.4	0.167	ν_21_ = −2.0	0.4	0.119
ν_21_ = −0.75	0.5	0.342		0.5	0.224	ν_22_ = 1.0	0.5	0.140
ν_22_ = −0.5	0.6	0.451		0.6	0.264		0.6	0.167
ν_23_ = 0.5	0.7	0.505		0.7	0.321		0.7	0.189
N = 10,000	0.8	0.601	N = 10,000	0.8	0.367	N = 10,000	0.8	0.217
	0.9	0.681		0.9	0.457		0.9	0.219

The solution  of  is, using Slutsky’s Law, a consistent estimator  of the population parameter  . Doing this for each non-diagonal element of  , we estimate the underlying non-diagonal elements of the full correlation matrix  . The diagonal elements are equal to one by assumption. This yields a consistent estimator  of the error covariance matrix  . Confidence intervals can be found by the delta method. Fig. 1 shows the graph of the function  for the left grid in Table A1.

In Table 4 we presented the full correlation matrices calculated by SUOP and by cmp. In Table A2. below we present the in-between covariance matrix and the full covariance matrix for the five-year panel considered in Section 5.1. We notice the fact that the grid-wise observation causes a considerable loss of information. About 60% of the information is lost. The estimates are not based on structural assumptions like “random effects” or errors where the correlations are specific functions of the time difference. Actually, this result could be used to estimate and test specific functional specifications of the covariance matrix. From Table A2. it is already obvious that “random effects” are to be rejected for this panel structure because in that case, the non-diagonal elements would have to be roughly equal to each other.

**Table A2:** In-between and full covariance matrices for the five-year panel.

	In-between covariance matrix					Full covariance matrix				
	2009	2010	2011	2012	2013	2009	2010	2011	2012	2013
2009	0.6434					1.0000				
2010	0.4852	0.6292				0.8899	1.0000			
2011	0.4123	0.4841	0.6289			0.7984	0.8945	1.0000		
2012	0.3771	0.4210	0.4793	0.6277		0.7490	0.8133	0.8910	1.0000	
2013	0.3413	0.3725	0.4156	0.4944	0.6321	0.7064	0.7482	0.7970	0.9270	1.0000

### B Table B1 is complete

**Table B1:** Comparison of the parameter estimates and their s.e.’s for Ordered Probit, Method of Moments, and Maximum Likelihood.

McK-Z *R* ^2^	0.1489		0.1481		0.1154	
*Health Satisfaction*						
Health: AGE	−0.0695	0.0046	−0.0693	0.0048	−0.0651	0.0045
Health: AGE^2^	0.0006	0.00005	0.0006	0.00005	0.0006	0.00005
Health: D_FEMALE	−0.0182	0.0175	−0.0181	0.0174	−0.0134	0.0166
Health: D_SINGLE	−0.0514	0.0291	−0.0510	0.0293	−0.0570	0.0278
Health: D_SEPARATED	−0.1241	0.0240	−0.1238	0.0243	−0.1108	0.0230
Health: Ln_LABOURINC	0.0289	0.0025	0.0288	0.0026	0.0227	0.0022
Health: CHILDREN	0.0357	0.0123	0.0356	0.0126	0.0308	0.0114
Health: D_EAST	−0.1678	0.0201	−0.1670	0.0196	−0.1402	0.0193
Health: DISABLE	−0.7991	0.0272	−0.7971	0.0268	−0.5825	0.0232
Health: Cut point 1	−1.8277	0.0184	−1.8240	0.0191	−1.8163	0.0181
Health: Cut point 2	−1.1161	0.0131	−1.1094	0.0134	−1.1122	0.0130
Health: Cut point 3	−0.3432	0.0109	−0.3415	0.0109	−0.3399	0.0107
Health: Cut point 4	0.9468	0.0123	0.9442	0.0124	0.9409	0.0122
McK-Z *R* ^2^	0.0278		0.0278		0.0196	
*Sleep Satisfaction*						
Sleep: AGE	−0.0380	0.0041	−0.0381	0.0042	−0.0348	0.0041
Sleep: AGE^2^	0.0004	0.00004	0.0004	0.00004	0.0003	0.00004
Sleep: D. FEMALE	−0.1213	0.0172	−0.1213	0.0172	−0.1204	0.0164
Sleep: D. SINGLE	0.0508	0.0302	0.0505	0.0307	0.0594	0.0284
Sleep: D. SEPARATED	−0.0762	0.0253	−0.0762	0.0260	−0.0658	0.0238
Sleep: Ln(HH.LABOURINC)	0.0067	0.0021	0.0066	0.0020	0.0062	0.0018
Sleep: # of CHILDREN	0.0219	0.0121	0.0220	0.0122	0.0164	0.0113
Sleep: YEARS of EDUCATION	0.0316	0.0032	0.0315	0.0031	0.0117	0.0028
Sleep: D. EAST GERMANY	−0.1063	0.0199	−0.1065	0.0196	−0.0763	0.0192
Sleep: Cut point 1	−1.6918	0.0175	−1.6912	0.0174	−1.7036	0.0174
Sleep: Cut point 2	−0.9791	0.0123	−0.9800	0.0123	−0.9831	0.0123
Sleep: Cut point 3	−0.9251	0.0106	−0.2970	0.0106	−0.2940	0.0104
Sleep: Cut point 4	0.7286	0.0114	0.7269	0.0115	0.7150	0.0113
